# Discriminative Role of Interleukin-6, Neuropilin-1, and Amphiregulin for Cirrhosis in Patients with Chronic Hepatitis B Infection

**DOI:** 10.5152/tjg.2026.26143

**Published:** 2026-07-09

**Authors:** Fatma Karakoc Ozudogru, Banu Karaca, Nesrin Turker, Bahar Ormen, Sezgin Vatansever, Huriye Erbak Yılmaz, Furkan Oguzhan Karalar, Fulya Gunsar, Alper Sener

**Affiliations:** 1 Department of Infectious Diseases, Bakırçay University Çiğli Research and Training Hospital, İzmir, Türkiye; 2 Department of Infectious Diseases, Katip Çelebi University Atatürk Training and Research Hospital, İzmir, Türkiye; 3 Department of Gastroenterology, Katip Çelebi University Atatürk Research and Training Hospital, İzmir, Türkiye; 4 Department of Biochemistry, Katip Çelebi University Atatürk Research and Training Hospital, İzmir, Türkiye; 5 Department of Gastroenterology, Ege University Faculty of Medicine Hospital, İzmir, Türkiye

**Keywords:** Amphiregulin, chronic hepatitis B, cirrhosis, IL-6, neuropilin-1

## Abstract

**Background/Aims:**

The roles of inflammatory and fibrogenic biomarkers in chronic hepatitis B (CHB) infection and hepatitis B virus (HBV)-related cirrhosis remain incompletely understood. The current study investigated the diagnostic and discriminative ability of amphiregulin (AREG), interleukin-6 (IL-6), and neuropilin-1 (Nrp-1) as biomarkers of HBV-related cirrhosis.

**Materials and Methods:**

This cross-sectional analytical study included 70 patients with CHB (43 without cirrhosis and 27 with HBV-related cirrhosis) and 60 healthy controls. Clinical and laboratory data were obtained from hospital records. Serum IL-6, Nrp-1, and AREG levels collected during outpatient visits were measured using enzyme-linked immunosorbent assay. Group differences were analyzed using the Kruskal–Wallis test, discriminative performance was evaluated using receiver operating characteristic (ROC) analysis, and factors independently associated with cirrhosis were identifiedusing logistic regression.

**Results:**

IL-6 and Nrp-1 levels were higher in patients with noncirrhotic and HBV-related cirrhotic CHB than in healthy controls (*P* < .001), with no significant difference between the patient subgroups. AREG levels were lower in the overall CHB cohort than in controls (*P* < .001) but higher in patients with cirrhosis than in those without cirrhosis (*P* = .001). ROC analysis showed that AREG had moderate discriminative ability for HBV-related cirrhosis (area under the curve (AUC) = 0.749). The optimal Youden-derived cutoff value was 2.29 ng/mL, with a sensitivity of 74% and a specificity of 65%. In logistic regression, AREG was independently associated with HBV-related cirrhosis (*P* = .012). Addition of ln-transformed AREG to age- and sex-adjusted aspartate aminotransferase-to-platelet ratio index (APRI)- and Fibrosis-4 index (FIB-4)-based models increased the AUC from 0.923 to 0.970 and from 0.938 to 0.980, respectively.

**Conclusion:**

AREG may serve as a complementary biomarker with moderate discriminative value for HBV-related cirrhosis but is unlikely to be useful as a standalone diagnostic or prognostic marker. Although IL-6 and Nrp-1 reflected underlying inflammatory activity, their ability to distinguish cirrhosis was limited.

**Main Points**

- Interleukin-6 (IL-6) and neuropilin-1 (Nrp-1) levels were significantly higher in patients with noncirrhotic and cirrhotic chronic hepatitis B (CHB) than in healthy controls but did not differ between the 2 patient subgroups.
- Amphiregulin (AREG) levels were lower in the overall CHB cohort than in healthy controls but were significantly higher in patients with cirrhosis than in those without cirrhosis.
- AREG showed moderate discriminative performance for hepatitis B virus (HBV)-related cirrhosis (area under the curve (AUC) = 0.749). An optimal cutoff value of 2.29 ng/mL yielded a sensitivity of 74% and a specificity of 65%.
- Logistic regression analysis identified AREG as independently associated with HBV-related cirrhosis (*P* = .012).
- Addition of AREG to APRI- and FIB-4-based models improved diagnostic performance, increasing AUC values from 0.923 to 0.970 and from 0.938 to 0.980, respectively. These findings suggest that AREG may serve as a complementary biomarker rather than a standalone diagnostic marker.

## Introduction

Approximately 254 million people worldwide are infected with hepatitis B virus (HBV), which can progress to chronic hepatitis, cirrhosis, and hepatocellular carcinoma. Reliable noninvasive tools for identifying advanced fibrosis and cirrhosis remain needed.^1^^,^^2^ Chronic hepatitis B (CHB)-related fibrogenesis is driven by extracellular mediators, including growth factors and cytokines such as interleukin-1 beta (IL-1β) and tumor necrosis factor alpha.^3^

Neuropilin-1 (Nrp-1) is a transmembrane glycoprotein involved in multiple ligand–receptor interactions and has recently been investigated as a regulator of fibrotic diseases.^4^ Nrp-1 modulates several growth factor pathways implicated in liver fibrosis and associated vascular alterations.^5^ Recent experimental data also suggest that Nrp-1 contributes to hepatic stellate cell activation and fibrotic remodeling.^6^ In addition, hepatocyte Nrp-1 has been linked to the hepatocyte growth factor/c-Met receptor pathway and fibrosis severity, supporting Nrp-1 as a potential therapeutic target in liver fibrosis.^7^

Amphiregulin (AREG), a member of the epidermal growth factor family, is expressed in various epithelial cell types.^8^ Studies in patients with nonalcoholic steatohepatitis (NASH) have shown that AREG produced by regulatory T cells (Tregs) activates profibrotic transcriptional programs in hepatic stellate cells via epidermal growth factor receptor (EGFR) signaling and that deletion of AREG in Tregs attenuates NASH-induced liver fibrosis.^9^ AREG has also been shown to be upregulated in murine and human NASH and to induce fibrogenic activity in hepatic stellate cells through multiple mitogenic signaling pathways. These findings suggest that AREG antagonists may represent clinically useful antifibrotic agents in nonalcoholic fatty liver disease.^10^

IL-6, a major proinflammatory cytokine, contributes to acute-phase responses, chronic inflammation, and fibrogenesis. Recent cytokine profiling studies in chronic HBV infection have shown that IL-6 and related inflammatory mediators vary across clinical disease courses, including liver cirrhosis.^11^ Broader studies of liver fibrosis also suggest that IL-6 and other ILs play a role in inflammation-driven fibrogenesis and advanced liver disease.^12^

Although many studies have examined cytokines such as IL-6 in CHB-related fibrosis and cirrhosis,^11^^,^^12^ human data on Nrp-1 are largely limited to hepatocellular carcinoma, with no studies evaluating its association with the clinical course of CHB.^13^ Likewise, although AREG has been linked to fibrosis in NASH and hepatocellular carcinoma (HCC),^9^^,^^10^ no human clinical study has assessed AREG in relation to HBV-related cirrhosis. Current guidelines increasingly favor noninvasive biomarkers over invasive procedures for assessing and monitoring CHB activity.^14^^-^^17^

The current study aimedto investigate the diagnostic and discriminative utility of Nrp-1, AREG, and IL-6 for HBV-related cirrhosis in patients with chronic HBV infection.

## Materials and Methods

### Study Design

This cross-sectional analytical study was conducted between September 2024 and May 2025 in the infectious diseases and gastroenterology clinics of 2 tertiary university hospitals: İzmir Katip Çelebi University Atatürk Training and Research Hospital and Ege University Hospital. Patients diagnosed with CHB infection were classified into groups according to the European Association for the Study of the Liver (EASL) Clinical Practice Guidelines^16^ and the Asian Pacific Association for the Study of the Liver (APASL) Guidelines.^17^

The study groups were as follows:

Group 1: Patients with hepatitis B e antigen (HBeAg)-negative chronic HBV infection (inactive carrier phase) and patients without cirrhosis under follow-up without antiviral therapy were included.

Group 2: Patients diagnosed with HBV-related cirrhosis and receiving antiviral therapy for up to 3 years were included. HBV-related cirrhosis was defined based on documented clinical, laboratory, radiological, and/or elastographic evidence, including findings consistent with cirrhotic liver morphology, portal hypertension, splenomegaly or thrombocytopenia, decompensation, or elastographic findings when available.

Control group: This group comprised of healthy volunteers without a known history of chronic liver disease, including previously diagnosed nonalcoholic fatty liver disease (NAFLD)/NASH, and without current medication use or alcohol consumption Hepatic steatosis was not systematically assessed by imaging or elastography in the control group.

Patients younger than 18 years; pregnant or breastfeeding women; those coinfected with hepatitis C virus, hepatitis D virus, or human immunodeficiency virus; and individuals with malignancy and inflammatory rheumatologic diseases or those receiving immunotherapy—conditions that could alter cytokine levels—were excluded. Demographic characteristics, alcohol consumption, hepatotoxic drug use, presence of hepatic steatosis, antiviral treatment status and duration, laboratory findings, hepatitis serology, and molecular test results were retrieved from the electronic medical records of the hospital.

**Figure 1.**
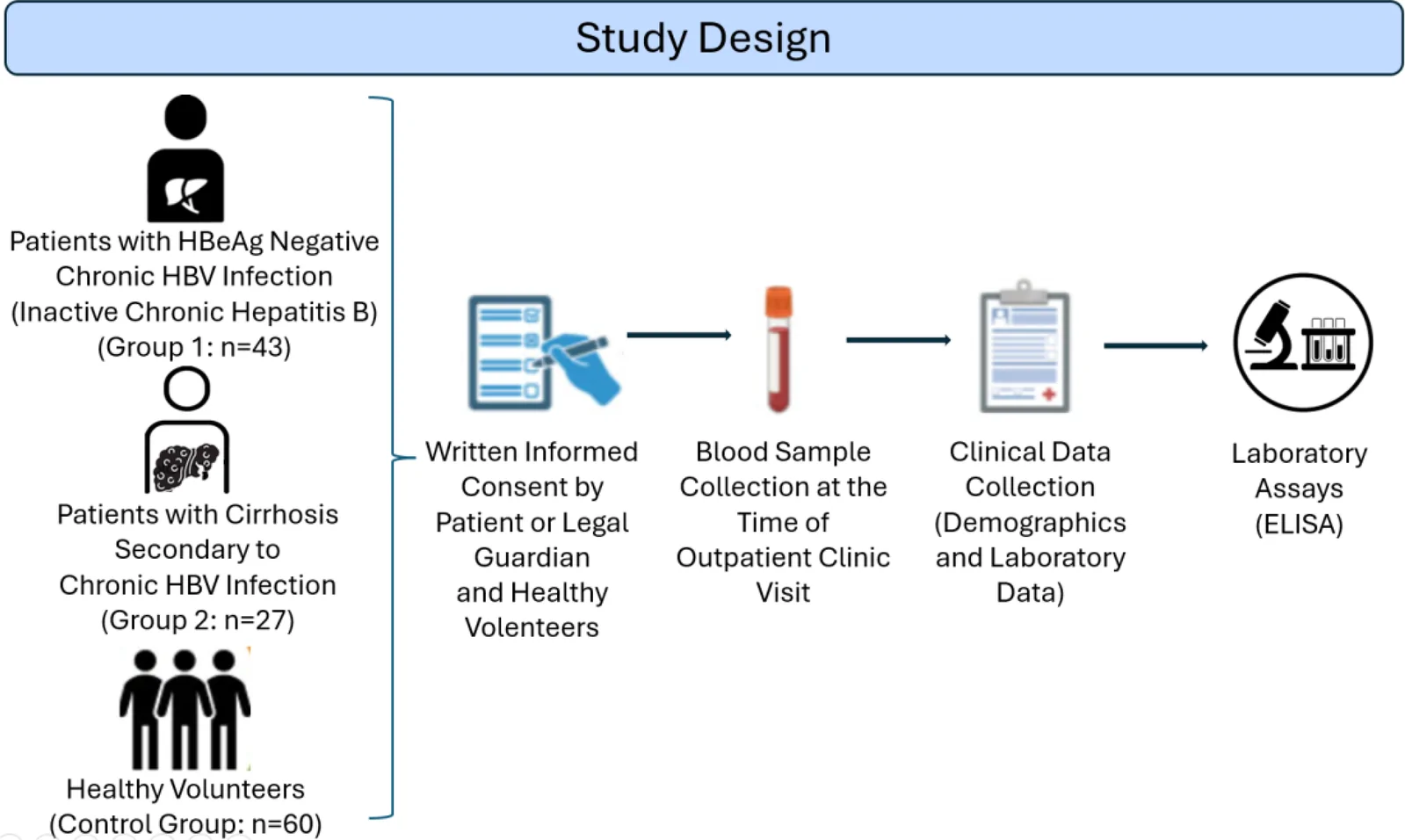
Study design.

The primary outcome was the discriminative performance of serum AREG, IL-6, and Nrp-1 for HBV-related cirrhosis. Secondary outcomes included differences in biomarker levels between patients and control, factors associated with cirrhosis, the optimal AREG cutoff value, and the incremental diagnostic value of AREG beyond APRI and FIB-4 (Figure 1).

Because no prior human studies had evaluated AREG in comparable HBV-related cirrhosis cohorts, a reliable a priori sample size calculation was not feasible. Therefore, this exploratory cross-sectional study included all eligible participants who met the inclusion criteria during the study period.

The study complied with the Declaration of Helsinki and was approved by the İzmir Katip Çelebi University Clinical Research Ethics Committee (No. 0019; July 18, 2024). Written informed consent was obtained from all participants. Generative artificial intelligence tools were used only for language editing and proofreading; all scientific content, data analysis, interpretation, and final text were reviewed and approved by the authors, who take full responsibility for the content of the manuscript.

### Measurement of Interleukin-6, Neuropilin-1, and Amphiregulin Levels

During outpatient visits, additional serum samples were collected from patients and controls. Samples were centrifuged at 4000 rpm for 10 minutes and stored at −80°C until analysis. Serum concentrations of IL-6, Nrp-1, and AREG were measured simultaneously in August 2025 using commercially available sandwich enzyme-linked immunosorbent assay (ELISA) kits according to the manufacturers’ instructions (Human Interleukin-6 ELISA kit, cat. no. E-EL-H6156, Elabscience Biotechnology, Houston, TX, USA; Human Neuropilin-1 ELISA kit, cat. no. E-EL-H6164, Elabscience Biotechnology, Houston, TX, USA; Human Amphiregulin ELISA kit, cat. no. 201-12-3148, Shanghai Sunred Biological Technology, Shanghai, China). Measurements were taken using a BioTek ELx800 microplate reader (BioTek Instruments, Inc., Winooski, VT, USA).

### Statistical Analysis

Normality of continuous variables was evaluated using histograms, probability plots, and Kolmogorov–Smirnov and Shapiro–Wilk tests with skewness-kurtosis measures. Descriptive data are presented as n (%) or mean ± SD. Two-group comparisons were performed using the independent samples *t*-test or Mann–Whitney *U*-test, as appropriate; comparisons among more than 2 groups were performed using the Kruskal–Wallis test with Bonferroni-adjusted Mann–Whitney *U* post hoc tests. Healthy controls and patients with CHB were compared with respect to demographic, clinical, and biomarker variables. Within the CHB cohort, factors associated with HBV-related cirrhosis were assessed. Logistic regression analysis was used to identify factors independently associated with cirrhosis. Variables with *P* < .05 in univariable analyses, together with clinically relevant covariates including age, IL-6, and Nrp-1, were included in the multivariable model. Because of the imbalance in age and sex between healthy controls and patients with CHB, additional age- and sex-adjusted analyses were performed. Biomarker levels were ln-transformed, and multivariable linear regression with robust standard errors was used, with results reported as geometric mean ratios (GMRs) and 95% CIs. The discriminative performance of biomarkers for cirrhosis was evaluated using receiver operating characteristic (ROC) curve analysis. AUC and 95% CIs were estimated using DeLong’s method, and optimal cutoff values were defined using the Youden index. APRI and FIB-4 scores were calculated using standard formulas: APRI = [(aspartate aminotransferase (AST)/upper limit of normal AST) / platelet count (10⁹/L)] × 100, and FIB-4 = [age × AST] / [platelet count (10^9^/L) × alanine aminotransferase (ALT)^0.5]. The incremental diagnostic value of AREG beyond APRI and FIB-4 was assessed using age- and sex-adjusted logistic regression models with and without ln(AREG). All analyses were performed using Statistical Package for the Social Sciences (SPSS), version 22.0 (IBM Corp., Armonk, NY, USA). A *P* value <.05 was considered statistically significant.

## Results

### Baseline Demographic and Clinical Characteristics

The study included 70 patients with CHB and 60 healthy controls. Group 1 consisted of 43 noncirrhotic patients with HBeAg-negative chronic HBV infection and group 2 included 27 patients with HBV-related cirrhosis. The mean age was 57.51 ± 11.73 years in group 1 and 62.67 ± 10.67 years in group 2. No significant differences in age or sex were observed between the groups (*P* =0.068 and *P* =0.333, respectively). The control group was significantly younger than the CHB cohort, as described below and addressed by age- and sex-adjusted analyses.

No illicit drug use or hepatotoxic medication exposure was recorded in either patient group. Alcohol consumption was significantly more frequent in group 2 than in group 1 (6/27 (22.2%) vs. 0/43 (0%), respectively; *P* = .001). However, detailed data on the amount and duration of alcohol consumption were not consistently available in the medical records; therefore, dose- or duration-based sensitivity analyses could not be performed. The prevalence of hepatic steatosis was numerically higher in group 2 than in group 1, although the difference was not statistically significant (21/27 (77.8%) vs. 29/43 (67.4%); *P* = .351). Baseline demographic and clinical characteristics are presented in Table 1.

### Serum Interleukin-6 and Neuropilin-1 Levels Are Increased, Whereas Amphiregulin Levels Are Lower in Hepatitis B e-Antigen-Negative Inactive Chronic Hepatitis B Infection and Chronic Hepatitis B–Related Cirrhosis

Kruskal–Wallis analysis showed significant differences in IL-6, Nrp-1, and AREG (all *P* < .0001) levels among the groups. In Bonferroni-adjusted post hoc Mann–Whitney *U*-tests, IL-6 and Nrp-1 levels were higher in both group 1 and group 2 than in controls (all *P* < .001) with no significant difference between the groups (*P* = 1.0 for both). Conversely, AREG levels were lower in group 1 (*P* < .001) and group 2 (*P* = .003) than in controls and also differed between group 1 and group 2 (*P* = .001) (Table 2; Supplementary Table 1).

Because the control group was significantly younger than the CHB cohort, age- and sex-adjusted analyses were performed using multivariable linear regression on ln-transformed biomarker levels. After adjustment for age and sex, IL-6 and Nrp-1 levels remained higher in group 1 and group 2 than in controls (IL-6 GMR: 6.65 and 7.51; Nrp-1 GMR: 2.23 and 2.26, respectively). In contrast, AREG levels remained lower in group 1 and group 2 than in controls (GMR: 0.25 and 0.47, respectively). AREG levels also remained significantly higher in group 2 than in group 1 after adjustment (GMR: 1.91). The direction and magnitude of these differences were consistent after adjustment, supporting the robustness of the patient–control biomarker comparisons (Supplementary Table 2).

Compared with controls (Mann–Whitney *U*-test), mean serum IL-6 levels were higher in group 1 (180.34 ± 410.93 pg/mL) and group 2 (201.33 ± 389.68 pg/mL) than in controls (56.52 ± 170.62 pg/mL; both *P* < .001). Similarly, mean Nrp-1 concentrations were significantly higher in group 1 (357.14 ± 93.98 pg/mL) and group 2 (355.75 ± 44.17 pg/mL) than in controls (170.78 ± 86.81 pg/mL; both *P* < .001). In contrast, mean AREG levels were significantly lower in group 1 (2.31 ± 1.45 ng/mL) and group 2 (5.24 ± 4.99 ng/mL) than in controls (9.56 ± 7.20 ng/mL; *P* < .001 and *P* = .003, respectively). Detailed subgroup estimates and corresponding *P* values are provided in Supplementary Table 3.

### Serum Amphiregulin Levels Are Significantly Higher in Hepatitis B Virus–Related Cirrhosis Than in Hepatitis B e-Antigen–Negative Chronic Hepatitis B Virus Infection

Patients with HBeAg-negative chronic HBV infection monitored without antiviral therapy (group 1) were compared with patients with HBV-related cirrhosis receiving antiviral therapy for up to longer than 3 years (group 2). Mean AREG levels were significantly higher in group 2 than in group 1 (*P* = .001).

To evaluate the discriminative performance of AREG for HBV-related cirrhosis, ROC curve analysis was performed, and the Youden index was used to identify the optimal exploratory cutoff value. The optimal Youden-derived AREG cutoff value was 2.29 ng/mL, corresponding to the highest Youden index (0.39). At this threshold, sensitivity was 74%, specificity was 65%, positive predictive value was 57%, and negative predictive value was 80%.

To assess the robustness of this cutoff value, additional clinically relevant thresholds were evaluated at fixed sensitivity and specificity levels. The threshold corresponding to 80% sensitivity was 1.79 ng/mL, with a specificity of 44%, whereas the threshold corresponding to 80% specificity was 3.35 ng/mL, with a sensitivity of 48%. These findings illustrate the expected trade-off between sensitivity and specificity and support interpreting the 2.29 ng/mL threshold as an exploratory discriminative cutoff value rather than a definitive clinical decision threshold. These analyses are shown in Supplementary Table 4.

### Receiver Operating Characteristic Analysis Demonstrates Moderate Discriminative Performance of Amphiregulin for Cirrhosis

**Figure 2.**
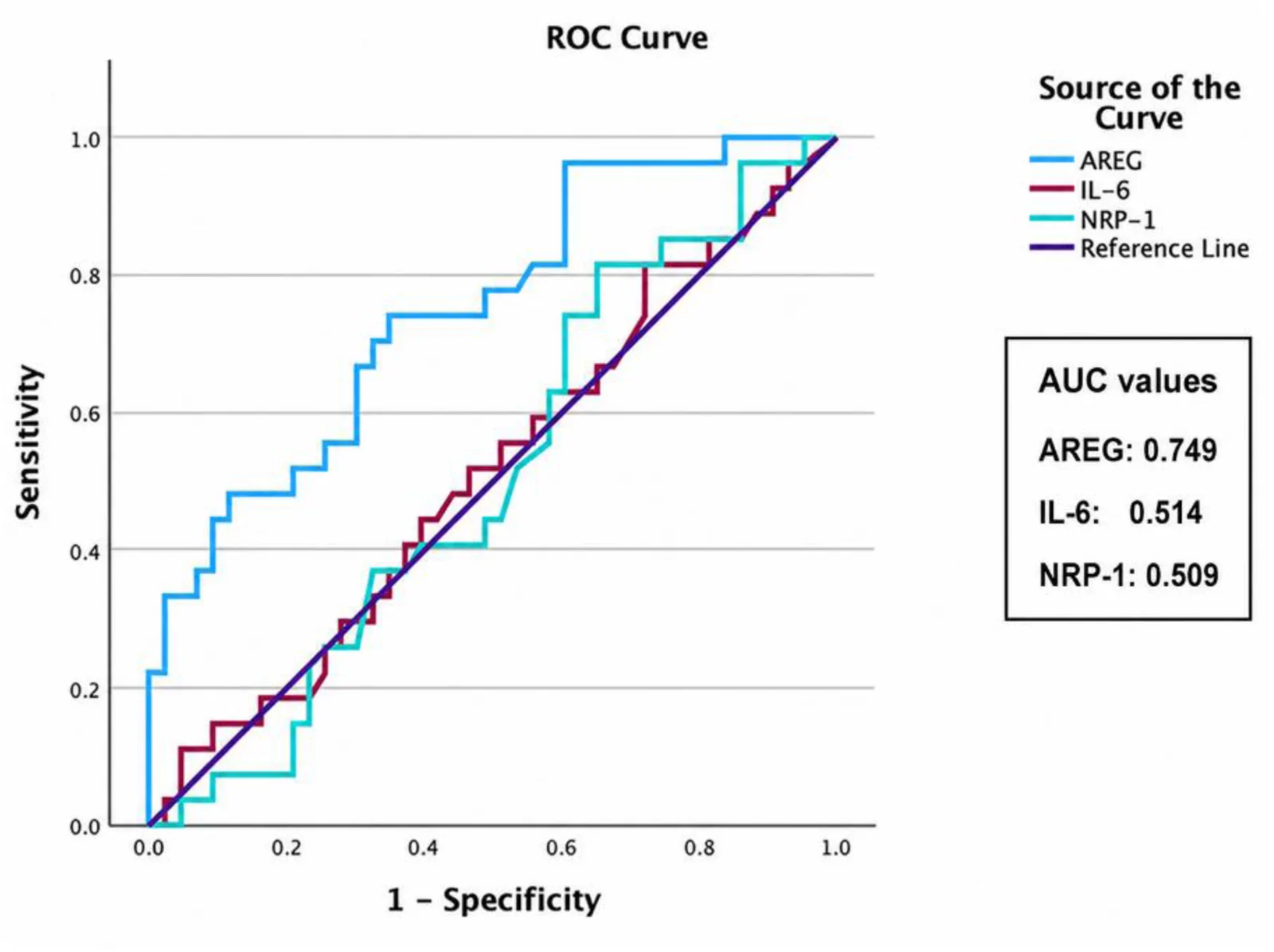
Receiver operating characteristic curves of interleukin-6 (IL-6), neuropilin-1 (Nrp-1), and amphiregulin (AREG) for discriminating hepatitis B virus (HBV)-related cirrhosis among patients with chronic HBV infection. AREG showed moderate discriminative ability, whereas IL-6 and Nrp-1 showed negligible discrimination. Area under the curve values are shown within the figure.

ROC curve analysis was performed to evaluate the discriminative ability of the investigated biomarkers for cirrhosis within the patient cohort. Among the biomarkers evaluated, AREG showed the highest AUC for discriminating cirrhosis within the patient cohort. AREG discriminated significantly between group 1 and group 2 (AUC = 0.749, standard error = 0.063; 95% CI: 0.626-0.872; *P* = .001). At the optimal cutoff value defined according to the highest Youden index, AREG yielded a sensitivity of 74.0% and a specificity of 65.0% (Figure 2).

In contrast, IL-6 and Nrp-1 levels did not differ significantly between the 2 patient groups (*P* = .833 and *P* = .943, respectively). ROC curve analyses demonstrated negligible diagnostic utility for cirrhosis discrimination (IL-6: AUC = 0.514, *P* = .497; Nrp-1: AUC = 0.509, *P* = .893).

### Comparison with Standard Noninvasive Scores

FIB-4 and APRI demonstrated excellent discrimination for cirrhosis within the patient cohort (AUC = 0.932 [95% CI: 0.857-1.000] and AUC = 0.898 [95% CI: 0.806-0.991], respectively), whereas MELD-Na and Child–Pugh demonstrated good discrimination (AUC = 0.840 [95% CI: 0.744-0.935] and AUC = 0.750 [95% CI: 0.652-0.848], respectively) (Supplementary Table 5). Notably, adding ln-transformed AREG to age- and sex-adjusted models containing APRI or FIB-4 significantly improved model fit and increased the AUC from 0.923 to 0.970 for the APRI-based model and from 0.938 to 0.980 for the FIB-4-based model (likelihood ratio test *P* < .001 and *P* = .002, respectively) (Supplementary Table 6).

### Serum Amphiregulin Level Is Independently Associated with Cirrhosis

Variables associated with cirrhosis were first evaluated using univariable logistic regression. Variables with *P* < .05 were included in the multivariable model. Age, IL-6, and Nrp-1 were also included a priori based on clinical/biological relevance and potential confounding.

In the univariable analysis, serum AREG level was significantly associated with cirrhosis (*P* = .004; 95% CI for Exp(B): 1.155-2.080). No significant associations were observed for age, IL-6, or Nrp-1 levels (*P* > .05) (Table 3).

In the multivariable logistic regression analysis, AREG remained independently associated with cirrhosis after adjustment for age, IL-6, and Nrp-1 (*P* = .008; 95% CI for Exp(B): 1.124-2.222). Age, IL-6, and Nrp-1 were not independently associated with cirrhosis (*P* > .05) (Table 4). These findings indicate that AREG was the only biomarker independently associated with cirrhosis in this study.

### Sensitivity Analyses for the Association Between Amphiregulin and Hepatitis B Virus–Related Cirrhosis

AREG remained significantly associated with cirrhosis after adjustment for age and sex, sex alone, and hepatic steatosis. The association also remained significant after excluding patients with recorded alcohol consumption. In sex- and hepatic steatosis-stratified analyses, the direction of the association was consistent, although CIs were wider in small subgroups. After adjustment for HBV viral load using log10 (HBV DNA + 1), the association was attenuated and no longer statistically significant. Among patients with cirrhosis with available data, antiviral treatment duration was not significantly correlated with AREG levels. Detailed information on the amount and duration of alcohol consumption was not consistently available; therefore, dose- or duration-based alcohol analyses could not be performed (Supplementary Table 7).

## Discussion

Fibrosis staging in chronic HBV infection is essential for prognosis and treatment planning, and noninvasive biomarkers may support earlier recognition of cirrhosis. In the current study, AREG levels were lower in the overall CHB cohort than in controls but higher in patients with HBV-related cirrhosis than in noncirrhotic CHB. AREG was also independently associated with cirrhosis, suggesting complementary discriminative value rather than longitudinal predictive value.

AREG, an EGFR ligand, is linked to inflammatory and neoplastic processes.^8^^,^^18^ Preclinical data suggest a context-dependent hepatic role, with hepatoprotective effects in acute injury and profibrotic effects during chronic damage, whereas human evidence in nonmalignant liver disease remains limited.^19^^-^^22^ Group 2 innate lymphoid cells (ILC2s) may support tissue repair through AREG-driven epithelial proliferation,^8^^,^^23^ and AREG can activate fibroblastic cells in liver fibrosis.^20^ However, the net effect of targeting the ILC2/AREG axis on injury resolution and fibrosis remains unclear.^23^ Only 2 animal studies have evaluated AREG in HBV infection,^24^^,^^25^ and no human data are available in chronic HBV infection or HBV-related cirrhosis.

APRI and FIB-4 scores are widely used for fibrosis risk stratification in chronic HBV.^14^^-^^17^ In the current cohort, both showed excellent cirrhosis discrimination and outperformed the investigational biomarkers (Supplementary Table 5). However, ln-transformed AREG improved model fit and AUC when added to age- and sex-adjusted APRI- and FIB-4-based models (Supplementary Table 6), suggesting complementary value.

AREG appears to have a context-dependent hepatic role. In early liver injury, it may act as a hepatoprotective and regenerative factor by reducing apoptosis and promoting compensatory proliferation through EGFR signaling. This may partly explain the higher baseline AREG levels observed in healthy controls than in patients with CHB.^8^^,^^19^

Serum AREG levels were lower in the overall HBV-infected cohort than in controls, but higher in cirrhotic than in noncirrhotic CHB patients. However, higher AREG levels in cirrhotic patients than in patients with inactive chronic HBV infection suggest a fibrosis-related increase within the CHB spectrum. Recent studies support a context-dependent role of AREG in liver injury, with hepatoprotective or regenerative effects in acute settings and profibrotic effects during chronic injury. Thus, AREG may function as a dual-purpose molecule: reduced circulating levels may reflect altered regenerative or inflammatory signaling in chronic HBV infection, whereas relatively higher levels in cirrhosis may reflect fibrosis-related EGFR signaling, hepatic stellate cell activation, and extracellular matrix remodeling. Therefore, higher AREG levels are associated with HBV-related cirrhosis rather than serving as a marker of longitudinal fibrotic progression.^9^^,^^10^^,^^20^^,^^26^

AREG levels were higher in patients with cirrhosis than in those with inactive chronic HBV infection. The optimal Youden-derived cutoff value for discriminating cirrhosis was 2.29 ng/mL, with an AUC of 0.749, indicating moderate discriminative ability.^27^ To the best of knowledge, no previous study has derived an AREG cutoff value for distinguishing HBV-related cirrhosis from noncirrhotic chronic HBV infection. In the logistic regression analysis, AREG remained independently associated with HBV-related cirrhosis, corresponding to a 1.581-fold increase in the odds of cirrhosis, supporting its potential role as a complementary biomarker.

Antiviral treatment may have influenced AREG levels by modifying HBV replication, necroinflammation, and cytokine/growth factor signaling. In the current cohort, antiviral exposure was present only in the cirrhotic group, making it difficult to distinguish disease-stage effects from treatment-related effects. Although antiviral treatment duration was not significantly correlated with AREG levels among patients cirrhosis with available data, this analysis was limited by sample size and incomplete treatment-duration data. Therefore, treatment-related confounding cannot be completely excluded and should be investigated in future longitudinal studies.

IL-6 has been associated with inflammation-driven fibrogenesis and advanced HBV-related liver disease.^11^^,^^12^ In the current study, IL-6 levels were higher in patients with CHB than in controls but did not differ significantly between patients with cirrhosis and those with inactive HBV infection. This finding may reflect the limited sample size, clinical heterogeneity, and other mediators involved in cirrhosis pathogenesis.^28^^-^^30^ Thus, although IL-6 appears to be involved in HBV-related fibrosis/cirrhosis, its standalone discriminative performance for cirrhosis may be limited, supporting further evaluation in larger multimarker studies.

Nrp-1 has been implicated in liver fibrosis and related vascular alterations.^4^^,^^5^^,^^31^ Although it may facilitate HBV entry,^31^ its role in chronic HBV infection and HBV-related cirrhosis remains unclear. In the current study, Nrp-1 levels were higher in both patient groups than in controls but did not differ between patient groups or discriminate cirrhosis. This finding may reflect limited cohort size and clinical heterogeneity. Further studies are warranted to clarify the role of Nrp-1 in HBV-related liver disease and cirrhosis discrimination.

In the current cohort, age, sex, and hepatic steatosis did not significantly different between the noncirrhotic CHB and HBV-related cirrhosis groups. Because these variables are recognized as important covariates in CHB-related fibrosis and liver outcome studies, their balance between groups may have reduced, although not eliminated, potential confounding.^32^^-^^34^ In addition, the higher frequency of alcohol use in the cirrhotic group was consistent with previous studies.^35^

The main limitations of the current study are its modest sample size and exploratory design, as no comparable human studies of AREG in HBV-related cirrhosis were available to support a reliable a priori sample size calculation. In addition, cirrhosis was defined according to documented clinical, laboratory, radiological, and/or elastographic findings rather than universal histological confirmation. Although this approach reflects routine clinical practice, the absence of histological confirmation for all patients may have introduced potential misclassification bias. Although the control group comprised volunteers without known chronic liver disease or alcohol use, NAFLD/NASH was not systematically excluded by imaging or elastography. Therefore, occult hepatic steatosis may have influenced biomarker comparisons between patient and control, particularly AREG levels. Healthy controls were younger because of strict eligibility criteria, introducing potential age-related bias. Although age- and sex-adjusted analyses were performed, statistical adjustment cannot fully compensate for the absence of design-stage matching when covariate overlap is limited and may result in unstable estimates. Future studies should use age- and sex-matched controls and/or propensity score–based designs in larger cohorts.^36^^-^^38^ Detailed information on the amount and duration of alcohol consumption was not consistently available in the medical records, precluding dose- or duration-based sensitivity analyses. In addition, biomarkers were measured at a single outpatient visit without serial follow-up, precluding assessment of temporal changes. Finally, the limited availability of human studies evaluating Nrp-1 and AREG in chronic HBV and HBV-related cirrhosis constrained direct comparisons with previous studies.

Despite these limitations, the current multicenter study had strict exclusion criteria to minimize potential confounding of cytokine and biomarker levels. The study included untreated HBeAg-negative patients with chronic HBV infection and patients with HBV-related cirrhosis who had received antiviral therapy for 3 years or less; however, treatment-related confounding cannot be excluded. To our knowledge, this is among the first human studies to evaluate Nrp-1 and AREG in HBV-related cirrhosis and to statistically derive an AREG cutoff value for discriminating cirrhosis in this population.

In conclusion, AREG showed moderate discriminative performance for HBV-related cirrhosis and remained independently associated with cirrhosis in logistic regression analyses. However, its performance was inferior to that of established noninvasive scores such as APRI and FIB-4. Therefore, AREG should be considered a complementary biomarker rather than a standalone diagnostic or prognostic tool. Consistent with current clinical guidelines^14^^-^^17^ emphasizing noninvasive assessment in chronic HBV management, AREG may provide additional biological information when evaluated together with established fibrosis indices. Larger prospective longitudinal studies are needed to validate these findings and to clarify the temporal relationship between AREG levels and changes in fibrosis stage.

## Supplementary Materials

Supplementary Material

## Figures and Tables

**Table 1.** Baseline Demographic and Clinical Characteristics of the Study Groups

Variable	Control Group (n = 60)	Group 1: Noncirrhotic CHB (n = 43)	Group 2: HBV-Related cirrhosis (n = 27)	P*
Age, years	27.22 ± 5.22	57.51 ± 11.73	62.67 ± 10.67	.068
Male sex, n (%)	23 (38.3)	22 (51.2)	17 (63.0)	.333
Alcohol consumption, n (%)	–	0 (0)	6 (22.2)	**.001**
Illicit drug use, n (%)	–	0 (0)	0 (0)	–
Hepatotoxic medication exposure, n (%)	–	0 (0)	0 (0)	–
Hepatic steatosis, n (%)	Not systematically assessed	29 (67.4)	21 (77.8)	.351

CHB, chronic hepatitis B; HBV, hepatitis B virus.

*P values refer to comparisons between group 1 and group 2. Bold values indicate statistically significant P values (P < .05).

**Table 2.** Comparison of IL-6, Nrp-1, and AREG Levels Between Patients with Chronic HBV Infection and Healthy Controls

Variable	Group	Mean	SD	*P*
IL-6* (pg/mL)	Chronic HBV infection (groups 1 and 2)	188.44	400.14	**<.001**
	Control group	56.52	170.62	
Nrp-1* (pg/mL)	Chronic HBV infection (groups 1 and 2)	356.61	78.18	**<.001**
	Control group	170.78	86.81	
AREG* (ng/mL)	Chronic HBV infection (groups 1 and 2)	3.44	3.56	**<.001**
	Control group	9.56	7.20	

AREG, amphiregulin; HBV, hepatitis B virus; IL-6, interleukin-6; Nrp-1, neuropilin-1.

*Based on the Mann–Whitney *U*-test.

Bold values indicate statistically significant *P* values (*P* < .05).

**Table 3.** Univariable Logistic Regression Analysis

Variable	*B*	SE	Wald	*P*	Exp(*B*)	95% CI	
						Lower	Upper
Age	0.042	0.024	3.211	.073	1.043	0.996	1.092
IL-6	0.000	0.001	0.046	.830	1.000	0.999	1.001
Nrp-1	0.000	0.003	0.005	.942	1.000	0.994	1.006
AREG	0.438	0.150	8.517	.004*	1.550	1.155	2.080

AREG, amphiregulin; *B*, regression coefficient; Exp(*B*), exponentiated regression coefficient (odds ratio); IL-6, interleukin-6; Nrp-1, neuropilin-1; SE, standard error.

*Bold values indicate statistically significant *P* values (*P* < .05).

**Table 4.** Multivariable Logistic Regression Analysis

Variable	*B*	SE	Wald	*P*	Exp(*B*)	95% CI	
						Lower	Upper
Age	0.031	0.028	1.193	.275	1.031	0.976	1.089
IL-6	0.000	0.001	0.461	.497	1.000	0.999	1.002
Nrp-1	0.000	0.003	0.018	.893	1.000	0.994	1.007
AREG	0.458	0.174	6.941	.008*	1.581	1.124	2.222

AREG, amphiregulin; *B*, regression coefficient; Exp(*B*), exponentiated regression coefficient (odds ratio); IL-6, interleukin-6; Nrp-1, neuropilin-1; SE, standard error.

*Bold values indicate statistically significant *P* values (*P* < .05).

## Data Availability

The data that support the findings of this study are available on request from the corresponding author.
