# Supplementary material for: Discriminative Role of Interleukin-6, Neuropilin-1, and Amphiregulin for Cirrhosis in Patients with Chronic Hepatitis B Infection
Source: Turk J Gastroenterol. 2026 Jul 9;37(9):985–94. doi: 10.5152/tjg.2026.26143 (PMC13554483; doi:10.5152/tjg.2026.26143)
Supplement: Supplementary file 1 [file supplementary_material.pdf]

Supplementary Table 1. Post hoc Bonferroni-corrected pairwise comparisons among study groups

Biomarker	Control vs Group 1	Control vs Group 2	Group 1 vs Group 2
IL-6*	*P* <.001	*P*<.001	*P*=1.0
Nrp-1*	*P* <.001	*P*<.001	*P*=1.0
AREG*	*P* <.001	*P*=.004	*P*=.001

Pairwise comparisons were performed using the Mann–Whitney U test with Bonferroni correction following a significant Kruskal–Wallis test. AREG, amphiregulin; Nrp-1, neuropilin-1; IL-6, interleukin-6.

Supplementary Table 2. Age- and Sex-Adjusted Comparisons of Serum Biomarker Levels Across Study Groups

Biomarker	Group 1 vs Control (GMR, 95% CI)	*P* -value	Group 2 vs Control (GMR, 95% CI)	*P* -value	Group 2 vs Group 1 (GMR, 95% CI)	*P* -value
IL-6 (pg/mL)	6.65 (2.08–21.26)	.001	7.51 (2.29–24.67)	<.001	1.13 (0.58–2.21)	.722
Nrp-1 (pg/mL)	2.23 (1.90–2.61)	<.001	2.26 (1.89–2.71)	<.001	1.02 (0.92–1.13)	.742
AREG (ng/mL)	0.25 (0.13–0.45)	<.001	0.47 (0.22–0.99)	.048	1.91 (1.32–2.76)	<.001

Biomarker concentrations were log-transformed and analyzed using multivariable linear regression adjusted for age and sex, with robust standard errors. Results are presented as geometric mean ratios (GMRs) with 95% confidence intervals (CIs) and *P* values. AREG, amphiregulin; CI, confidence interval; GMR, geometric mean ratio; Nrp-1, neuropilin-1; IL-6, interleukin-6.

Supplementary Table 3. Comparison of IL-6, Nrp-1, and AREG Levels Between Group 1,2 and the Control Group

Variable	Group	Mean	SD	*P*
IL-6* (pg/mL)	Group 1	180.34	410.93	<.001
	Control group	56.52	170.62	
Nrp-1* (pg/mL)	Group 1	357.14	93.98	<.001
	Control group	170.78	86.81	
AREG* (ng/mL)	Group 1	2.31	1.45	<.001
	Control group	9.56	7.20	
IL-6* (pg/mL)	Group 2	201.33	389.68	<.001
	Control group	56.52	170.62	
Nrp-1* (pg/mL)	Group 2	355.75	44.17	<.001
	Control group	170.78	86.81	
AREG* (ng/mL)	Group 2	5.24	4.99	=.003
	Control group	9.56	7.20	

P -values are based on the Mann–Whitney U test. AREG, amphiregulin; Nrp-1, neuropilin-1; IL-6, interleukin-6.

Supplementary Table 4. Diagnostic Performance of AREG for Discriminating Cirrhosis

Criterion	Cutoff (ng/mL)	Sensitivity (%)	Specificity (%)	PPV (%)	NPV (%)
Highest Youden Index (0.39)	2.29	74	65	57.1	80.0
80% sensitivity	1.79	80	44	47.8	79.2
80% specificity	3.35	48	80	70.6	71.7

AREG, amphiregulin; NPV, negative predictive value; PPV, positive predictive value. The optimal cutoff was defined by the highest Youden index.

Supplementary Table 5. Diagnostic Performance of Experimental Biomarkers and Standard Clinical Scoring Systems for Discriminating Cirrhosis Within the Patient Cohort (Group 1 vs Group 2)

Variable	AUC (95% CI)	*P* -value	Optimal cutoff*	Sensitivity (%)	Specificity (%)
AREG	0.749 (0.631–0.866)	<.001	2.29	74	65
IL-6	0.514 (0.373–0.655)	.497	23.05	81	28
Nrp-1	0.509 (0.372–0.647)	.893	340.04	81	35
APRI	0.898 (0.806–0.991)	<.001	0.60	77	98
FIB-4	0.932 (0.857–1.000)	<.001	2.39	81	100
Child-Pugh	0.750 (0.652–0.848)	<.001	6.00	50	100
MELD-Na	0.840 (0.744–0.935)	<.001	9.00	81	71

*Optimal cutoff values were derived by maximizing the Youden index (sensitivity + specificity − 1). AUC, area under the curve; AREG, amphiregulin; APRI, AST to platelet ratio index; FIB-4, fibrosis-4 index; IL-6, interleukin-6; MELD-Na, Model for End-Stage Liver Disease–Sodium; Nrp-1, neuropilin-1.

Supplementary Table 6. Incremental Diagnostic Value of AREG Beyond APRI and FIB-4 for discriminating cirrhosis (Age- and Sex-Adjusted Logistic Regression Models)

Model	AREG adjusted OR*	95% CI		Model AUC (95% CI)	Likelihood ratio test *P* †
Age + sex + APRI				0.923 (0.852–0.994)	
Age + sex + APRI + ln(AREG)	7.73	1.82–32.84	0.006	0.970 (0.936–1.000)	<0.001
Age + sex + FIB-4				0.938 (0.860–1.000)	
Age + sex + FIB-4 + ln(AREG)	5.99	1.45–24.77	0.013	0.980 (0.954–1.000)	0.002

AREG, amphiregulin; APRI, aspartate aminotransferase-to-platelet ratio index; AUC, area under the receiver operating characteristic curve; CI, confidence interval; FIB-4, fibrosis-4 index; OR, odds ratio.
*ln(AREG) denotes the natural log transformation; odds ratios are reported per doubling of AREG (derived from the ln-transformed term).
† Likelihood-ratio test (LRT) *P* values compare nested models with vs without ln(AREG). AUC 95% CIs were calculated using DeLong's method.

Supplementary Table 7. Sensitivity analyses for the association between AREG and HBV-related cirrhosis

Analysis	Model / restriction	n	Cirrhosis events	AREG effect	95% CI	*P*	Interpretation
Main analysis	Logistic regression: cirrhosis ~ ln(AREG)	70	27	2.8	1.53–5.13	.001	Consistent with main association
Age- and sex-adjusted	ln(AREG) + age + sex	70	27	2.87	1.50–5.49	.001	Association remained significant
Sex-adjusted	ln(AREG) + sex	70	27	3.03	1.60–5.73	.001	Association remained significant
Hepatic steatosis-adjusted	ln(AREG) + hepatic steatosis	70	27	2.9	1.52–5.51	.001	Association remained significant
HBV viral load-adjusted	ln(AREG) + log10(HBV DNA + 1)	70	27	1.61	0.76–3.42	.21	Same direction, but attenuated after adjustment
Alcohol sensitivity	Alcohol users excluded; cirrhosis ~ ln(AREG)	64	21	3.08	1.55–6.12	.001	Association remained significant after excluding alcohol users
Alcohol sensitivity, adjusted	Alcohol users excluded; ln(AREG) + age + sex	64	21	3.09	1.49–6.40	.002	Association remained significant
Male subgroup	Men only; cirrhosis ~ ln(AREG)	39	17	2.54	1.12–5.73	.025	Association remained significant
Female subgroup	Women only; cirrhosis ~ ln(AREG)	31	10	3.88	1.33–11.33	.013	Association remained significant
No hepatic steatosis subgroup	No steatosis; cirrhosis ~ ln(AREG)	20	6	6.89	1.03–46.10	.046	Wide CI due to small subgroup
Hepatic steatosis subgroup	Steatosis present; cirrhosis ~ ln(AREG)	50	21	2.42	1.22–4.80	.011	Association remained significant
Antiviral treatment duration	Spearman correlation between AREG and antiviral duration among cirrhotic patients with available data	12	12	rho=0.21	—	.518	No significant correlation; duration recorded in years

AREG was ln-transformed for logistic regression analyses. Odds ratios are reported per doubling of AREG. HBV viral load was entered as log10(HBV DNA + 1). Alcohol use was recorded only among cirrhotic patients; therefore, sensitivity analysis was performed by excluding patients with recorded alcohol consumption. Detailed data on the amount and duration of alcohol use were not consistently available, precluding dose- or duration-based alcohol analyses. Antiviral treatment duration was evaluated only among cirrhotic patients with available data because antiviral exposure was structurally related to group allocation. AREG, amphiregulin; CI, confidence interval.
